# Association of small dense low-density lipoprotein with cardiovascular outcome in patients with coronary artery disease and diabetes: a prospective, observational cohort study

**DOI:** 10.1186/s12933-020-01015-6

**Published:** 2020-04-03

**Authors:** Jing-Lu Jin, Hui-Wen Zhang, Ye-Xuan Cao, Hui-Hui Liu, Qi Hua, Yan-Fang Li, Yan Zhang, Na-Qiong Wu, Cheng-Gang Zhu, Rui-Xia Xu, Ying Gao, Xiao-Lin Li, Chuan-Jue Cui, Geng Liu, Jing Sun, Qian Dong, Yuan-Lin Guo, Jian-Jun Li

**Affiliations:** 1 State Key Laboratory of Cardiovascular Disease, Fu Wai Hospital, National Center for Cardiovascular Diseases, Chinese Academy of Medical Sciences, Peking Union Medical College, BeiLiShi Road 167, Beijing, 100037 China; 2 Department of Cardiology, Xuanwu Hospital, Capital Medical University, Beijing, 100053 China; 3 Department of Cardiology, Beijing Anzhen Hospital, Capital Medical University, Beijing, 100029 China

**Keywords:** sdLDL, MACEs, CAD, DM

## Abstract

**Background:**

Elevation in small dense low-density lipoprotein (sdLDL) is common in patients with diabetes mellitus (DM), which has already been reported to be associated with incidence of coronary artery disease (CAD). The aim of the present study was to investigate the prognostic value of plasma sdLDL level in patients with stable CAD and DM.

**Methods:**

A total of 4148 consecutive patients with stable CAD were prospectively enrolled into the study and followed up for major cardiovascular events (MACEs) up to 8.5 years. Plasma sdLDL level was measured in each patient by a direct method using automated chemistry analyzer. The patients were subsequently divided into four groups by the quartiles of sdLDL and the association of sdLDL level with MACEs in different status of glucose metabolism [DM, Pre-DM, normal glycaemia regulation (NGR)] was evaluated.

**Results:**

A total of 464 MACEs were documented. Both Kaplan–Meier analysis and Cox regression analysis indicated that the patients in quartile 4 but not quartile 2 or 3 of sdLDL level had significantly higher rate of MACEs than that in lowest quartile. When the prognostic value of high sdLDL was assessed in different glucose metabolism status, the results showed that the high sdLDL plus DM was associated with worse outcome after adjustment of confounding risk factors (hazard ratio: 1.83, 95% confident interval: 1.24–2.70, p < 0.05). However, no significant association was observed for high sdLDL plus Pre-DM or NGR.

**Conclusions:**

The present study firstly indicated that elevated levels of plasma sdLDL were associated with increased risk of MACEs among DM patients with proven CAD, suggesting that sdLDL may be useful for CAD risk stratification in DM.

## Background

It has been well recognized that the low-density lipoprotein (LDL)-cholesterol (LDL-C) is one of the major risk factors of coronary artery disease (CAD). According to recent guidelines, achieving lower levels of LDL-C for patients with very high risk has been highly recommended [1, 2]. However, among patients who reached the appropriate LDL-C targets, the risk of incident cardiovascular events remains high [3]. In fact, LDL particles are heterogeneous in size and density and can be categorized into large, buoyant LDL (lbLDL) and small, dense LDL (sdLDL) [4]. A study performed by Hoogeveen et al. in 2014 indicated that the sdLDL but not lbLDL was positively associated with the presence of CAD in Atherosclerosis Risk in Communities (ARIC) study [5].

In hypertriglyceridemic states, excess triglyceride (TG) in LDL is hydrolysised by hepatic lipase (HL) and converts to sdLDL [6]. Compared with lbLDL, the particles of sdLDL are more atherogenic. The particles of sdLDL have smaller size, higher penetration into the arterial wall, lower binding affinity for the LDL receptor, longer plasma residence time, and increased susceptibility to oxidation [7–10]. In some studies on the relationship between lipid and other CAD risk markers, data suggested that sdLDL was more significantly associated with inflammatory markers than LDL-C [11]. In the cross-sectional study by Koba and his colleague, sdLDL levels were more powerful than LDL-C levels for the determination of CAD severity [12]. In two large observational cohort studies, sdLDL could predict the incident CAD among individuals with low cardiovascular risk according to their LDL-C levels [5, 13]. Unfortunately, among secondary prevention patients, the role of sdLDL in the progression of atherosclerotic cardiovascular disease (ASCVD) was less determined.

Diabetes mellitus (DM) is also one of the most important risk factors of ASCVD and can cause bad prognosis in patients with established CAD [14, 15]. In hyperglycemic status, the HL-mediated pathway is more active [6]. Thus, the plasma level of sdLDL is significantly elevated in pre-diabetes (Pre-DM) and DM individuals [13, 16]. Furthermore, sdLDL is more susceptible to glycation than lbLDL, which may increase its atherogenicity [17]. However, the impact of high sdLDL on cardiovascular outcome in DM patients was not yet clarified. Hence, the aim of the present study was to investigate the prognostic value sdLDL on major cardiovascular events (MACEs) in patients with stable CAD under different glucose metabolism status.

## Materials and methods

### Study population

Our study complied with the Declaration of Helsinki and was approved by the local ethical review board. Informed written consents were obtained from all patients enrolled in this study.

The details were described in the flowchart (Additional file 1: Figure S1). From March 2011 to December 2016, 5132 patients who were admitted to three medical centers and diagnosed as angiography-proven CAD (coronary stenosis ≥ 50% in at least one of main coronary arteries). 984 patients did not enter the final analysis. 233 did not agree to enter the program and others were for excluded for following criteria: with missing data, acute coronary syndrome (ACS), decompensated heart failure, severe liver and/or renal insufficiency, thyroid dysfunction, systematic inflammatory disease, and malignant disease. Patients in the analyses were diagnosed as stable CAD because of typical symptoms of transient angina. Patients were followed-up by telephone or face to face interviewing at 6 months’ intervals by experienced nurses or physicians. The MACEs were defined as cardiovascular mortality, non-fatal myocardial infarction (MI), stroke, unplanned percutaneous coronary intervention (PCI) or coronary artery bypass grafting (CABG), and hospitalized unstable angina. Non-fatal myocardial infarction was diagnosed as positive cardiac enzymes along with typical chest pain or electrocardiogram serial changes. Stroke was diagnosed by medical history, typical symptoms, and characterized imaging.

DM was diagnosed by fasting plasma glucose (FPG) ≥ 7.0 mmol/L or the 2-h plasma glucose of the oral glucose tolerance test ≥ 11.1 mmol/L, haemoglobin A1c (HbA1c) level ≥ 6.5% or currently using anti-diabetic drugs or insulin. Pre-DM was diagnosed in participants who did not have diagnosed DM but met the American Diabetes Association (ADA) criteria of Pre-DM [fasting plasma glucose 5.6 to 6.9 mmol/L, 2-h glucose ranging from 7.8 to 11.0 mmol/L, or hemoglobin A1c (HbA1c) level from 5.7 to 6.4% [18]. Patients who were with neither DM nor pre-DM were defined as normal glucose regulation (NGR). Hypertension was diagnosed as medical history of hypertension, currently receiving antihypertensive drugs or hospital recorded systolic blood pressure (SBP) ≥ 140 mmHg and/or diastolic blood pressure (DBP) ≥ 90 mmHg for three or more consecutive times. The body mass index (BMI) was calculated as weight (kg) divided by height (m) squared. Patients who had smoking habit of at least 1 cigarette per day on admission were classified as current smokers. Those who reported alcohol intake at least once a week were sorted as drinking. Family history of CAD was defined when myocardial ischemia or infarction was documented in at least one first-degree relative. Baseline medications (medications before admission) were collected by interviewing or from hospital-recorded medical history. Patients who received statin therapy at the time of discharging from hospital were also recorded.

### Laboratory analysis

Fasting blood samples were obtained from each patient after 12-h fasting once upon admission. Blood samples were collected into EDTA-containing tube. After centrifugation at 3000 rpm for 10 min at 4 °C, plasma was collected and stored at − 80 °C. Plasma concentrations of total cholesterol (TC), TG, LDL-C and HDL-C were measured by automatic biochemistry analyzer (Hitachi 7150, Tokyo, Japan) in an enzymatic assay. Non-high density lipoprotein cholesterol (Non-HDL-C) was calculated as TC minus HDL-C. Plasma levels of sdLDL were measured by an automated homogeneous assay (DENKA SEIKEN CO., LTD, Tokyo, Japan) [19]. The measured sdLDL was with calibration range of 0.0–100 mg/dL. The concentrations of glucose were measured by enzymatic hexokinase method. HbA1c was measured using Tosoh Automated Glycohemoglobin Analyser (HLC-723G8, Tokyo, Japan).

### Evaluation of CAD severity

Angiographic data were collected from catheter laboratory records. The procedure was performed by three experienced interventional physicians. The angiographic results were evaluated by same physicians and the Gensini score (GS) which was calculated by assigning a severity score to each coronary lesion according to the degree of stenosis and the significance of location, was used to present the extent of coronary severity according our previous study. The precise method of calculating GS was introduced by Gensini GG in 1983 [20].

### Statistical analysis

The values for the continuous variables and the categorical variables were presented as the mean ± SD, median (Q1–Q3 quartiles) or number (percentage). The Kolmogorov–Smirnov test was used to test the distribution pattern. The differences of variables among groups were analyzed using Student t-test, analysis of variance, or nonparametric test where appropriate. The Kaplan–Meier method was used to estimate the event-free survival rates among groups. The log-rank test was used to test the statistical significance. The hazard ratios (HRs) and 95% confidence intervals (CI) were calculated by univariate and multivariate Cox regression analyses. A p-value < 0.05 was considered statistically significant. The statistical analyses were performed with SPSS version 21.0 software (SPSS Inc., Chicago, IL, USA).

## Results

### Baseline characteristics

As shown in Table 1, patients were divided into four groups by the quartile value of sdLDL (Q1: sdLDL < 18.30 mg/dL, Q2: 18.30 ≤ sdLDL < 26.70 mg/dL, Q3: 26.70 ≤ sdLDL < 38.58 mg/dL, Q4: sdLDL ≥ 38.58 mg/dL). Patients with higher sdLDL were with younger age and more likely to be female (p < 0.001). The body mass index (BMI), glucose, HbA1c, TC, LDL-C, TG increased while the proportions of family history of CAD, and statin use decreased according to levels of sdLDL (p < 0.001). Patients with sdLDL Q4 had the highest proportion of hypertension, highest GS and the lowest proportion of NGR while those with sdLDL Q3 had the highest proportion of Pre-DM and lowest level of HDL-C. No significant difference was found regarding, drinking, smoking, creatinine and other medications among the four groups of patients.

**Table 1:** Baseline characteristics

Variables	Total	sdLDL Q1	sdLDL Q2	sdLDL Q3	sdLDL Q4	p
	n = 4148	n = 1036	n = 1028	n = 1035	n = 1049	
Clinical characteristics						
Age, years	59.7 ± 9.8	58.4 ± 9.0	57.5 ± 8.8	56.5 ± 8.8	56.0 ± 8.9	< 0.001
Male, n (%)	2967 (71.5)	748 (72.2)	747 (72.7)	760 (73.4)	712 (67.9)	0.022
BMI (kg/m^2^)	26.0 ± 3.1	25.5 ± 3.1	25.8 ± 3.2	26.1 ± 2.9	26.5 ± 3.0	< 0.001
Hypertension, n (%)	2686 (64.8)	649 (62.6)	672 (65.4)	661 (63.9)	704 (67.1)	0.164
Glucose metabolism status, n (%)						0.001
NGR	892 (21.5)	272 (26.3)	211 (20.5)	211 (20.4)	198 (18.9)	
Pre-DM	1702 (41.0)	407 (39.3)	413 (40.2)	447 (43.2)	435 (41.5)	
DM	1554 (37.5)	357 (34.5)	404 (39.3)	377 (36.4)	416 (39.7)	
Family history of CAD, n (%)	594 (14.3)	129 (12.5)	141 (13.7)	149 (14.4)	175 (16.7)	0.045
Current smoker, n (%)	2284 (55.1)	578 (55.8)	563 (54.8)	565 (54.6)	578 (55.1)	0.95
Drinking, n (%)	1376 (33.2)	352 (34.1)	330 (32.1)	352 (34.0)	341 (32.5)	0.694
Laboratory findings						
Glucose (mmol/L)	6.2 ± 2.0	5.8 ± 1.7	6.2 ± 2.1	6.2 ± 2.0	6.5 ± 2.2	< 0.001
HbA1c (%)	6.5 ± 1.2	6.4 ± 1.1	6.6 ± 1.3	6.5 ± 1.2	6.6 ± 1.3	< 0.001
Creatinine (μmol)	77.3 ± 15.2	77.6 ± 15.9	78.2 ± 15.6	77.4 ± 15.3	76.8 ± 15.6	0.544
TC (mmol/L)	4.12 ± 1.08	3.24 ± 0.74	3.80 ± 0.69	4.22 ± 0.74	5.19 ± 1.02	< 0.001
HDL-C (mmol/L)	1.05 ± 0.29	1.08 ± 0.30	1.06 ± 0.30	1.02 ± 0.28	1.04 ± 0.27	< 0.001
LDL-C (mmol/L)	2.50 ± 0.94	1.79 ± 0.64	2.26 ± 0.59	2.62 ± 0.69	3.34 ± 1.00	< 0.001
Non-HDL-C (mmol/L)	3.07 ± 1.04	2.15 ± 0.67	2.74 ± 0.59	3.20 ± 0.64	4.15 ± 0.97	< 0.001
sdLDL (mg/dL)	30.18 ± 16.14	13.42 ± 3.25	22.39 ± 2.36	32.20 ± 3.47	52.37 ± 13.02	< 0.001
TG (mmol/L)	1.50 (1.10–2.12)	1.07 (0.87–1.33)	1.37 (1.09–1.74)	1.67 (1.32–2.19)	2.24 (1.74–2.98)	< 0.001
Gensini score	22 (10–38)	22 (10–37)	22 (11–36)	24 (13–38)	24 (13–41)	0.047
Medications						
Statins at baseline, n (%)	2645 (63.8)	732 (70.7)	690 (67.1)	370 (64.3)	491 (53.2)	< 0.001
Statins at discharge, n (%)	3989 (96.2)	1020 (98.5)	991 (96.4)	986 (95.3)	992 (94.6)	< 0.001
Aspirin at baseline, n (%)	2505 (60.4)	617 (59.6)	601 (58.5)	633 (61.2)	654 (62.3)	0.280
ACEIs/ARBs at baseline, n (%)	1143 (27.6)	271 (26.2)	297 (28.9)	293 (28.3)	282 (26.9)	0.482
β-blockers at baseline, n (%)	2202 (53.1)	551 (53.2)	542 (52.7)	562 (54.3)	547 (52.1)	0.333
Antidiabetic drugs at baseline						
OADs, n (%)	815 (19.6)	217 (20.9)	192 (18.7)	185 (17.9)	223 (21.1)	0.163
Insulin, n (%)	462 (11.1)	122 (11.8)	100 (9.7)	126 (12.2)	114 (10.9)	0.297

Data were expressed as mean ± SD, median with 25th and 75th percentile or n (%)

*BMI* body mass index, *NGR* normal glucose regulation, *Pre-DM* pre-diabetes mellitus, *DM* diabetes mellitus, *HbA1c* haemoglobin A1c, *TC* total cholesterol, *TG* triglyceride, *LDL-C* low density lipoprotein cholesterol, *HDL-C* high density lipoprotein cholesterol, *GS* Gensini score, *ACEIs* ACE inhibitors, *ARBs* angiotensin receptor blockers, *OADs* oral antidiabetes drugs

### sdLDL levels and cardiovascular outcomes

During a median follow-up time of 5.1 years (interquartile range: 3.9 to 5.9 years), 464 (11.2%) of 4148 patients had MACEs (70 cardiovascular deaths, 49 suffered non-fatal MI, 108 had non-fatal strokes and 158 received unplanned revascularization and 79 suffered hospitalized unstable angina). The unadjusted cumulative incidence curves for MACEs by sdLDL quartiles are shown in Additional file 1: Figure S2. Setting sdLDL Q1 as reference, patients with sdLDL in the highest quartile (Q4) showed significantly higher rate of events while no difference was observed for those with sdLDL Q2 and Q3. In Cox proportional hazard regression analyses, according to univariate and multivariate models, patients with sdLDL Q4 presented 1.32-fold and 1.38-fold higher risk of MACEs than those with sdLDL Q1, respectively (Table 2). In the meanwhile, quartiles of LDL-C and non-HDL-C were not associated with MACEs (Additional file 1: Table S1).

**Table 2:** Cox regression analysis according to quartiles of sdLDL

sdLDL quartile (n, events/subjects)	HR (95% CI)	
	Unadjusted	Full adjusted
Q1 (106/1036)	Ref.	Ref.
Q2 (109/1028)	1.04 (0.79–1.35)	1.04 (0.80–1.37)
Q3 (108/1035)	1.01 (0.77–1.32)	1.05 (0.80–1.38)
Q4 (141/1049)	*1.32 (1.02–1.69)	*1.38 (1.03–1.84)

Model was adjusted for age, sex, body mass index, smoking, hypertension, diabetic status, family history of coronary artery disease, Gensini score, high density lipoprotein cholesterol, triglyceride and baseline statins

*p < 0.05

**Fig. 1:**
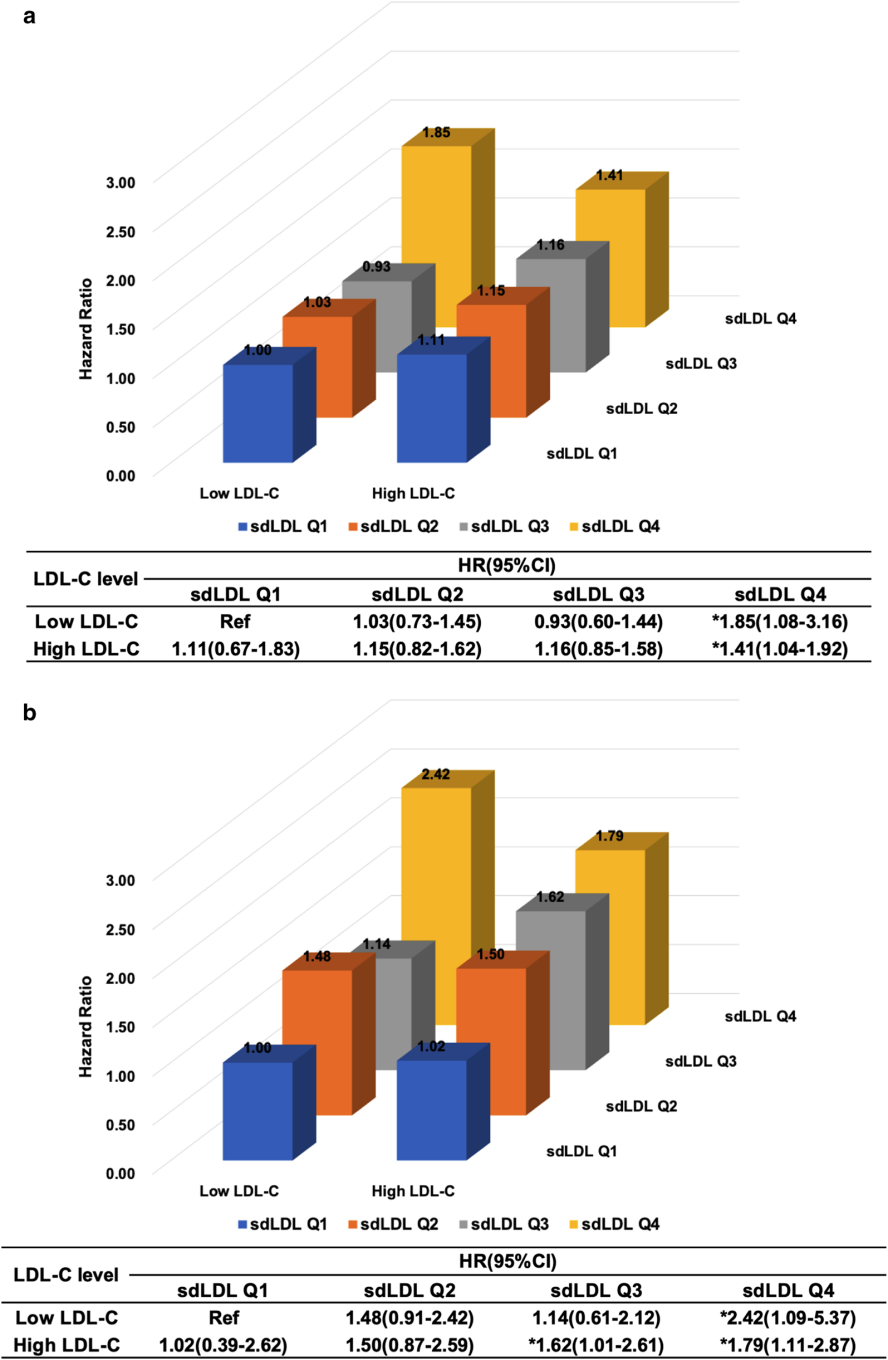
Adjusted hazard ratios for incident MACEs by sdLDL-C quartiles stratified by LDL-C risk categories in **a** total population and **b** DM group. Model adjusted for age, sex, body mass index, smoking, hypertension, diabetic status, family history of coronary artery disease, Gensini score, high density lipoprotein cholesterol, triglyceride and baseline statins * for p < 0.05

We further investigated the association of sdLDL-C with the risk of MACEs when patients were stratified by high and low LDL-C. In total population, both sdLDLQ4 plus low LDL-C and sdLDLQ4 plus high LDL-C were predictive of MACEs (Fig. 1a). Similar results were observed in DM patients (Fig. 1b). In separate low and high LDL-C groups, sdLDLQ4 was associated with 2.14-fold and 2.11-fold higher risk of cardiovascular risk respectively (p < 0.05, Additional file 1: Table S2). There appeared significant interaction between sdLDL and LDL-C in predicting MACEs (Additional file 1: Table S2, p for interaction = 0.018).

### sdLDL levels, different glucose metabolism status and cardiovascular outcomes

As presented in Additional file 1: Figure S3a, both DM and Pre-DM groups had higher levels of plasma sdLDL than NGR group, but no significant difference existed between DM and Pre-DM. Furthermore, higher proportion of patients were with high sdLDL in DM and Pre-DM than that in NGR (Additional file 1: Figure S3b). When NGR, Pre-DM and DM groups were each further divided into four subgroups by the quartile value of sdLDL, similar patterns of sdLDL distribution were also observed in Pre-DM and DM while higher percentage of patients were with sdLDL Q1 and Q2 in NGR (Additional file 1: Figure S3c).

**Fig. 2:**
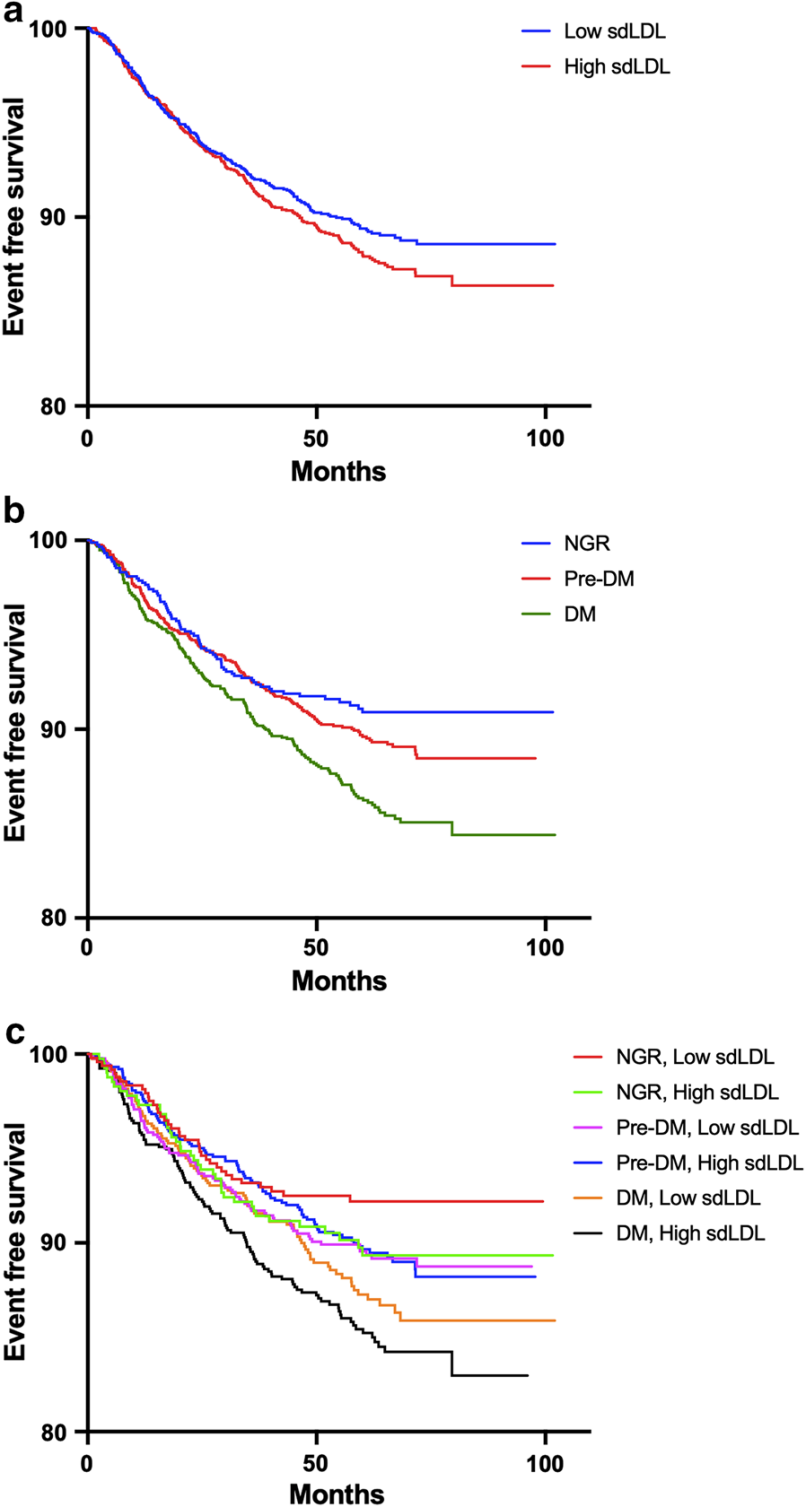
Kaplan–Meier curves according to **a** median value of sdLDL **b** different glucose metabolism status **c** both status

**Fig. 3:**
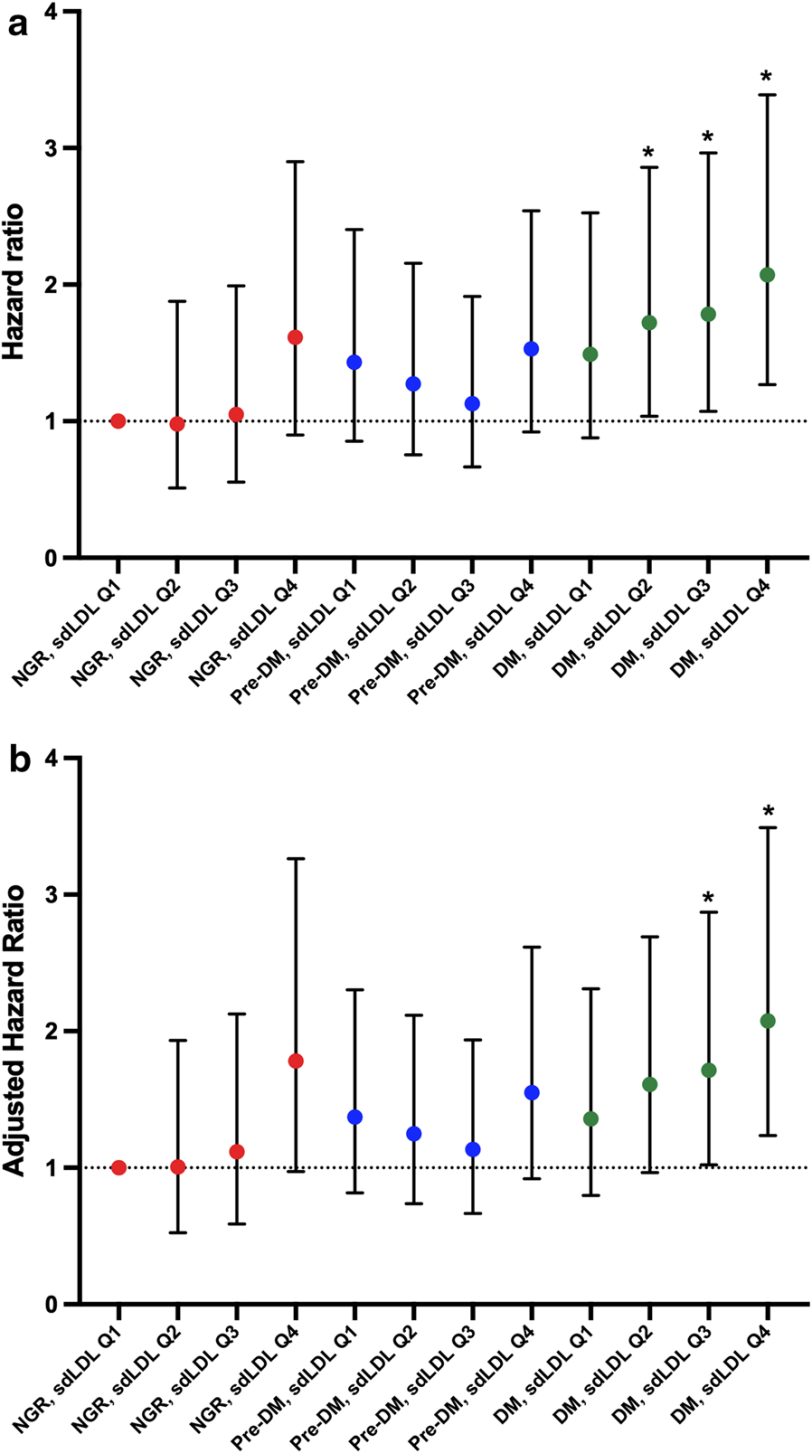
Unadjusted (**a**) and adjusted (**b**) Cox Model according to quartile value of sdLDL and glucose metabolism status. Model adjusted for age, sex, body mass index, smoking, hypertension, diabetic status, family history of coronary artery disease, Gensini score, high density lipoprotein cholesterol, triglyceride and baseline statins. *p < 0.05

In Kaplan–Meier analysis, high sdLDL did not present higher events rate than low sdLDL (log rank p > 0.05, Fig. 2a). According to different glucose metabolism status, DM but not Pre-DM showed higher risk of worse prognosis than NGR (log rank p < 0.001, Fig. 2b). In subgroups by both status, individuals with DM plus high sdLDL had the highest rate of MACEs (log rank p = 0.004, Fig. 2c). When the prognostic value of high sdLDL in different glucose metabolism status was evaluated by Cox models, both low and high sdLDL plus DM had higher risk of MACEs but high sdLDL plus DM was associated with the worst outcome (HR: 1.83, 95% CI 1.24–2.70, p < 0.05, Table 3). No significant association was observed for high sdLDL in Pre-DM and NGR (p > 0.05). This association was further evaluated in 12 groups by the quartile value of sdLDL and glucose metabolism status (Fig. 3a, b). After adjustment by traditional risk factors, patients in DM plus sdLDL Q3 and DM plus sdLDL Q4 but not those in DM plus Q1 and DM plus Q2 subgroups presented higher risk of cardiovascular events. Significant interaction between the quartiles sdLDL and diabetic status was observed in predicting MACEs (Additional file 1: Table S2, p for interaction < 0.001).

**Table 3:** sdLDL levels in relation to cardiovascular events in patients with different glucose metabolism status

sdLDL	HR (95% CI)		
	Events/subjects 464/4148	Crude model	Adjusted model
NGR			
Low sdLDL	37/483	Ref.	Ref.
High sdLDL	41/409	1.33 (0.85–2.08)	1.36 (0.87–2.15)
Pre-DM			
Low sdLDL	86/820	1.36 (0.93–2.01)	1.30 (0.88–1.91)
High sdLDL	92/882	1.34 (0.91–1.96)	1.29 (0.87–1.91)
DM			
Low sdLDL	92/761	*1.63 (1.11–2.38)	*1.49 (1.01–2.19)
High sdLDL	116/793	**1.95 (1.35–2.83)	**1.83 (1.24–2.70)

Model was adjusted for age, sex, body mass index, smoking, hypertension, family history of coronary artery disease, Gensini score, high density lipoprotein cholesterol, triglyceride and baseline statins

* p < 0.05

** p < 0.01

## Discussion

Increased plasma levels of sdLDL was one of the key features of diabetic dyslipidemia [21]. Although the link of sdLDL and presence of ASCVD has been well established in previous community-based studies, the exact relationship between sdLDL and cardiovascular outcome in ASCVD patients with NGR, Pre-DM and DM was not yet examined [5, 13]. In this prospective study of stable CAD patients, we tried to investigate the prognostic value of sdLDL in patients with different metabolism status. The main findings of our study were as follows: (1) In overall population, the highest quartile of sdLDL conveyed 1.38-fold higher risk of MACEs. Similar associations between sdLDL and prognosis were also observed among individuals with high and low LDL-C levels. (2) In patients with different glucose metabolism status, more significant risk of MACEs was identified in those with combined status of high sdLDL plus DM. To the best of our knowledge, this is the first study which addressed the increased atherogenicity of sdLDL in DM by a large populational-based observational cohort.

Currently, according to existing guidelines, routine measurement of plasma LDL-C level was crucial in the prevention of ASCVD [1, 2]. Previous study reported that there was 21% proportional reduction in the incidence of MACEs per mmol/L LDL-C reduction [22]. In fact, LDL particles were a collection of heterogeneous particles which had different features in size, density, and lipid composition [4]. With the same plasma LDL-C concentration, individuals who were with more sdLDL particles might have higher numbers of LDL particles. In the study by Otvos et al. in 2011, they found that in the individuals with low LDL-C but high LDL particle number (LDL-P), which reflected higher levels of sdLDL, the event free survival rate was significantly lower than those with low LDL-P but high LDL-C [23]. Furthermore, previous study suggested that sdLDL had a higher propensity to bind to receptors on endothelium [10]. Hence, the inter-individual variation in sdLDL levels may partly explain the high event rate among individuals who received optimal lipid-lowering therapy and reached LDL-C target. Therefore, identifying whether high sdLDL is a potential impedimental factor for risk reduction was crucial, especially in patients with CAD and DM [2].

A great deal of evidence indicated that the sdLDL was related to the incidence of atherosclerotic disease [5, 13, 24]. Several studies in different populations using various sdLDL measurements suggested that the sdLDL was related to the carotid intima thickness and in association with progression of carotid artery plaque [25–27]. Wu et al. reported in 2019 that elevated sdLDL concentration was observed in ACS patients (n = 121) but not in healthy controls (n = 172) [28]. In ARIC study (11419 community-based participants and followed-up for 11 years), the plasma sdLDL was measured by an automated homogeneous assay. They found that the individuals with sdLDL ≥ 75th percentile presented 1.51-fold higher risk of CAD than those in the lowest quartile [5]. And also, sdLDL-C coud predict the risk for the incidence of CAD among those who were classified as low cardiovascular risk by their LDL-C levels. Similar result was reported for normoglycemic individuals in the Multi-Ethnic Study of Atherosclerosis (MESA) [13].

Besides, several studies reported the association of sdLDL and the degree of coronary stenosis in patients with established CAD. In a study by Koba et al. in 2006, results showed that plasma sdLDL levels, which determined by heparin–magnesium precipitation, increased with the increment of diseased coronary vessels or GS [12]. They also reported that sdLDL levels were superior to LDL-C levels in predicting coronary severity [29]. In the Atherothrombosis Intervention in Metabolic Syndrome with Low HDL/High Triglycerides and Impact on Global Health Outcomes (AIM-HIGH) trial, which enrolled 3094 CAD patients, data indicated that sdLDL was not predictive of cardiovascular outcome [30]. The result of the present study, which was conducted in a large Chinese cohort of stable CAD patients, indicated that participants with sdLDL Q4 had the higher risk of MACEs despite their LDL-C levels. The predictive value of sdLDL was even stronger than LDL-C and non-HDL-C in our study population. This finding, therefore, may provide novel evidence for the risk reduction in ASCVD patients who had achieved their LDL-C target.

The number of patients with DM in China continued to increase in the past few years. Recently, the morbidity rate was reported to be more than 10% in Chinese populations [31]. In both European Society of Cardiology/European Atherosclerosis Society (ESC/EAS) and American College of Cardiology/American Heart Association (ACC/AHA) guidelines, patients with DM and ASCVD were defined as very high risk individuals and intensive LDL-C lowering treatment was highly recommended [1, 2]. Proprotein convertase subtilisin/kexin type 9 (PCSK9) inhibitor considerably decreased LDL-C and could be useful in reducing the risk of events in patients with DM and ASCVD receiving maximally tolerated statin [32, 33]. However, routine lipid measures may not well reflect the compositional changes of lipid parameters in DM and Pre-DM patients. For example, no significant increase in LDL-C levels was found in some DM and Pre-DM patients [16]. In patients with metabolic disorder, the increase in very low-density lipoprotein (VLDL) particles could initiate a sequence of hydrolysis process and result in higher plasma sdLDL levels [6]. According to the study by Peradze et al. in 2019, treatment of obese patients with liraglutide could induce reductions in number of sdLDL [34]. Data from MESA study showed that the plasma levels of sdLDL were largely elevated in patients with impaired glucose metabolism when they were with similar LDL-C as NGR [13]. In agreement with these previous findings, our study also showed that there were higher sdLDL levels in individuals with Pre-DM and DM. However, in the MESA study, for participants with impaired fasting glucose or type 2 DM (T2DM), sdLDL did not convey a significant risk. Interestingly, in our study, high plasma sdLDL brought 1.83-fold higher risk of MACEs when patients were combined with DM, but no such association was observed in ones with Pre-DM and NGR. Notably, it is worth mentioning that this is the first study to report the positive prognosis of sdLDL in DM. Compared with MESA study, our study had several strengths. Firstly, we selected patients with angiography-proven CAD as the study population. Secondly, the MESA study included only 1048 patients with impaired fasting glucose (IFG) or DM and compared them with NGR population. Moreover, IFG is an intermediate state between NGR and DM. In our present study, high sdLDL presented higher risk of MACEs in DM but not in those with mild impaired glucose metabolism (Pre-DM). Hence, classifying patients with DM and IFG into the same group might contribute to the null finding. Thirdly, in our study, the numbers of patients and events with DM (208 events of 1554 patients) or Pre-DM (178 events of 1702 patients) exceeded those in MESA (84 events of 1048 patients). Last but not least, this was a prospective, real-world study. Therefore, the current study might provide novel information about the relationship of sdLDL and cardiovascular outcomes in DM patients.

One of the limitations of this study was that the level of sdLDL was measured only once at baseline. Furthermore, although the Cox models were adjusted by multiple traditional risk factors, the possible effect of other confounders could not be fully eliminated. Patterns of follow-up statin use were not provided due to unavailable data. Finally, we did not assess all the metabolic factors for those with DM or Pre-DM due to the features of patients in the present study.

## Conclusions

In conclusion, this large sample size and long-term follow-up study, for the first time, indicated that elevated plasma sdLDL could predict worse outcomes in patients with CAD and DM but not in those with NGR or Pre-DM. These novel findings may extend the knowledge about the prognostic value of sdLDL particle in DM patients.

## Supplementary information

**Additional file 1: Table S1.** Cox regression analysis according to quartiles of LDL-C and non-HDL-C. **Table S2.** Predictive value of sdLDL in different LDL-C levels and glucose metabolism status. **Figure S1.** Flowchart of the study. **Figure S2.** Cumulative incidence of MACEs according to different sdLDL levels. **Figure S3.** Continuous (a) and category (b to c) sdLDL levels according to different glucose metabolism status.

## Data Availability

The datasets used and/or analyzed during the current study are not publicly available but are available from the corresponding author on reasonable request.

## Abbreviations

NGR: Normal glucose regulation
Pre-DM: Pre-diabetes
DM: Diabetes mellitus
CAD: Coronary artery disease
ASCVD: Atherosclerotic cardiovascular disease
MACEs: Major adverse cardiovascular events
PCI: Percutaneous coronary artery intervention
CABG: Coronary artery bypass grafting
ACS: Acute coronary syndrome
BMI: Body mass index
HbA1c: Haemoglobin A1c
TC: Total cholesterol
TG: Triglyceride
LDL-C: Low density lipoprotein cholesterol
HDL-C: High density lipoprotein cholesterol
non-HDL-C: Non-high density lipoprotein cholesterol
sdLDL: Small dense low density lipoprotein
GS: Gensini score
