# Supplementary material for: Association of small dense low-density lipoprotein with cardiovascular outcome in patients with coronary artery disease and diabetes: a prospective, observational cohort study
Source: Cardiovasc Diabetol. 2020 Apr 3;19:45. doi: 10.1186/s12933-020-01015-6 (PMC7118853; doi:10.1186/s12933-020-01015-6)

**Additional file 1**

**Table S1** Cox regression analysis according to quartiles of LDL-C and non-HDL-C

| **Variable** | **HR (95%CI)** | | | |
| --- | --- | --- | --- | --- |
|  | **sdLDL Q1** | **sdLDL Q2** | **sdLDL Q3** | **sdLDL Q4** |
| **LDL-C** |  |  |  |  |
| Unadjusted | Ref | 0.93(0.71-1.23) | 1.10(0.84-1.44) | 1.23(0.93-1.62) |
| Adjusted | Ref | 0.95(0.72-1.24) | 1.11(0.85-1.44) | 1.23(0.95-1.60) |
| **Non-HDL-C** |  |  |  |  |
| Unadjusted | Ref | 1.13(0.86-1.49) | 1.23(0.93-1.64) | 1.32(0.98-1.79) |
| Adjusted | Ref | 1.13(0.86-1.49) | 1.21(0.92-1.58) | 1.30(0.99-1.69) |

Model was adjusted for age, sex, body mass index, smoking, hypertension, diabetic status, family history of coronary artery disease, Gensini score, high density lipoprotein cholesterol, triglyceride and baseline statins

**Table S2** Predictive value of sdLDL in different LDL-C levels and glucose metabolism status

| **Subgroup** | **HR (95%CI)** | | | | **p _int_** |
| --- | --- | --- | --- | --- | --- |
|  | **sdLDL Q1** | **sdLDL Q2** | **sdLDL Q3** | **sdLDL Q4** |  |
| **LDL-C level** |  |  |  |  | 0.018 |
| **Low LDL-C** | Ref | 0.81(0.56-1.17) | 1.03(0.67-1.58) | *2.14(1.22-3.76) |  |
| **High LDL-C** | Ref | 1.16(0.63-2.11) | 1.39(0.78-2.47) | *2.11(1.14-3.59) |  |
| **Diabetic Status** |  |  |  |  | <0.001 |
| **NGR** | Ref | 0.97(0.50-1.87) | 1.07(0.55-2.09) | 1.57(0.78-3.17) |  |
| **Pre-DM** | Ref | 0.97(0.63-1.50) | 0.92(0.58-1.43) | 1.27(0.80-2.02) |  |
| **DM** | Ref | 1.12(0.79-1.82) | 1.09(0.71-1.67) | *1.62 (1.09-2.41) |  |

Model was adjusted for age, sex, body mass index, smoking, hypertension, diabetic status (for LDL-C subgroups), family history of coronary artery disease, Gensini score, high density lipoprotein cholesterol, triglyceride and baseline statins

* for p<0.05; p_int_ means p for interaction

**Figure S1** Flowchart of the study

**
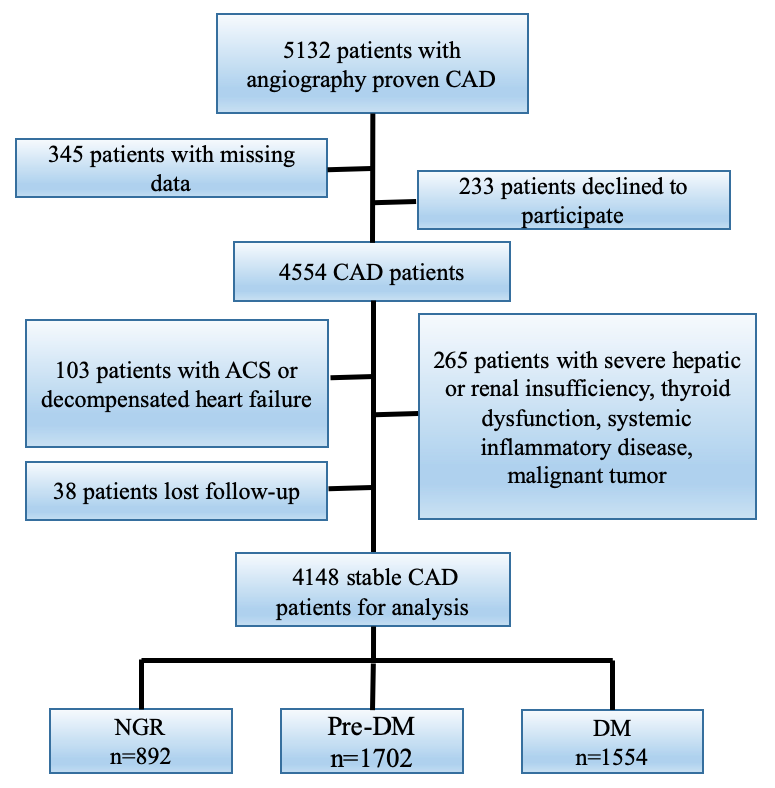
**

**Figure S2** Cumulative incidence of MACEs according to different sdLDL levels

**Figure S3** Countinuous (a) and category (b to c) sdLDL levels according to different glucose metabolism status


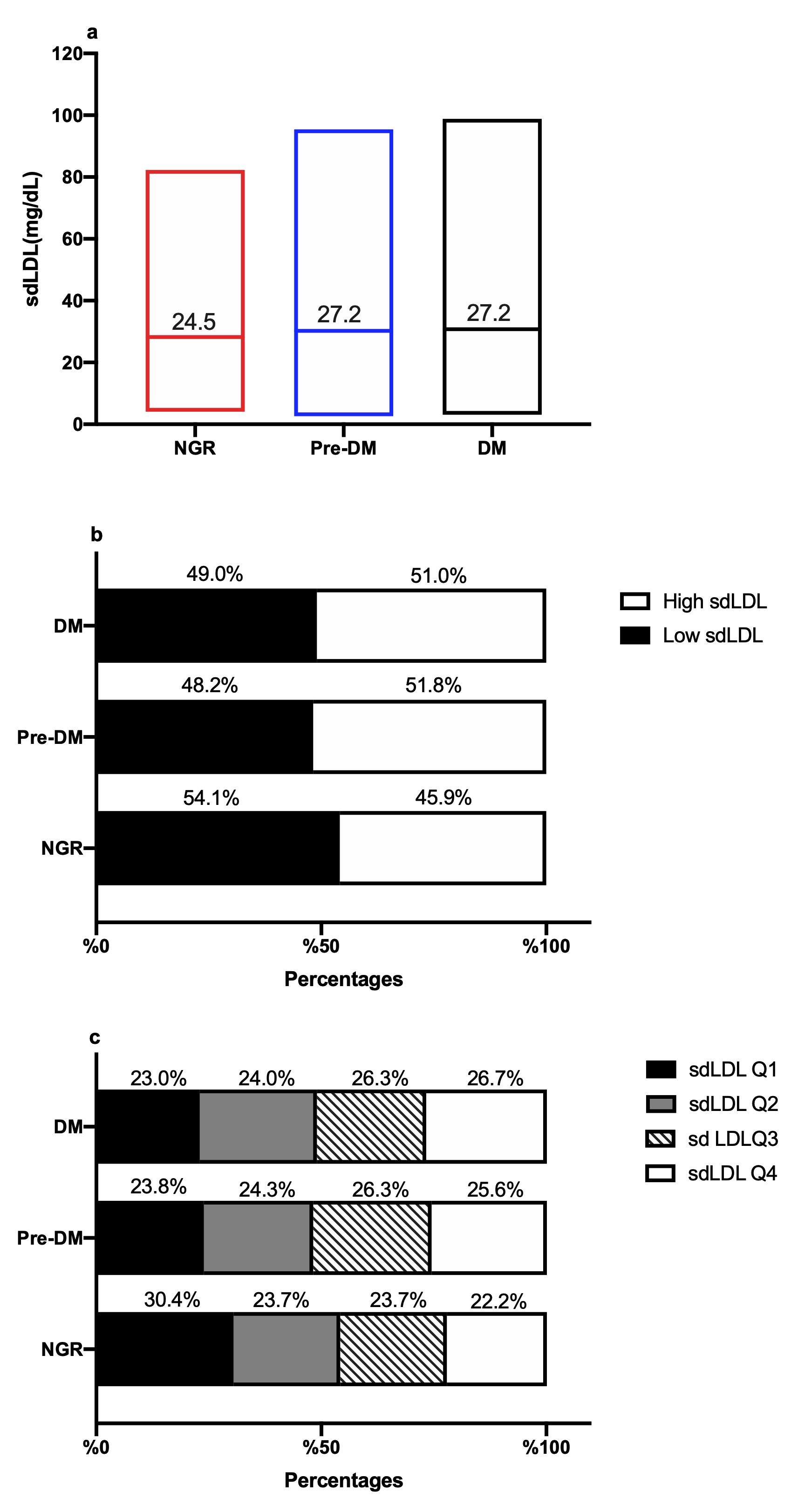

Supplement: Supplementary file 1 — Additional file 1: Table S1. Cox regression analysis according to quartiles of LDL-C and non-HDL-C. Table S2. Predictive value of sdLDL in different LDL-C levels and glucose metabolism status. Figure S1. Flowchart of the study. Figure S2. Cumulative incidence of MACEs according to different sdLDL levels. Figure S3. Continuous (a) and category (b to c) sdLDL levels according to different glucose metabolism status. [file 12933_2020_1015_MOESM1_ESM.docx]
